# False Aneurism of the Femoral Artery, the Sequence of Abscess

**Published:** 1853-02

**Authors:** S. S. Stratford

**Affiliations:** Toronto


From the Canada Medical Journal.

*False Aneurism of the Femoral Artery, the Sequence of Abscess—cured by Ligature upon the External Iliac.* By S. S. Stratford, M.R.C.S.L., Toronto.

In the Spring of 1845, I was called by Dr. Corbin to see a son of Mr. Leek, a wealthy farmer residing in the township of Oxford East, C.W. The boy was about 15 years of age, and was affected with a very large abscess in the right thigh; the whole thigh was greatly swollen, especially its anterior and internal portions, which swelling extended from the groin to the ham. The skin was red, shining, and pitted upon pressure, while an examination distinctly indicated a fluctuation. It had been progressing for a month or six weeks, notwithstanding the means used to arrest it, and now distinctly presented all the indications of a very large and extensive abscess.

I had been called to consult as to the propriety of opening the abscess, and immediately decided as to its absolute necessity. Accordingly, I made an opening in a dependent position upon the inside of the thigh, about its centre—good healthy-looking pus freely escaped, amounting to about a pint—not a drop of blood was lost, and, to ascertain the extent of the abscess, I introduced a large probe, upwards, as high as the groin, and downwards nearly to the ham; a tent was introduced into the opening, and a bandage applied round the limb. Upon calling about a week afterwards, I found that the matter which had collected from time to time had been regularly evacuated, and was now but small in quantity; the general swelling of the limb was greatly diminished, and only a thickening remained, which I expected would gradually be absorbed, and the patient would get well.

To my surprise, however, in about six weeks' time, I received a message from Dr. Corbin, saying that matter had again collected in the thigh, and he wished that I would come over and open it once more. Accordingly, I went to see the patient, and found the thigh greatly swelled; the swelling occupied exactly the same position which the original had previously done; it extended from the groin to the ham. The skin appeared red and shining, but did not pit upon pressure; there was distinctly a fluctuation to be observed; there was something about the swelling that did not present to my mind the distinct idea of an abscess, but that it was an aneurism certainly did not enter into my imagination; there was not the least pulsation present, while its recent history certainly misled both myself and the Doctor into the belief that the swelling was the sac of an abscess again refilled with pus; the consequence was, that we decided upon puncturing the swelling. I selected a spot that I thought the most prominent and likely to make a good opening for the exit of matter, but after introducing the lancet a considerable depth, I found that no pus was evacuated, which created my surprised. Dr. Corbin then took the lancet and introduced it into another spot, and out flowed a stream of arterial blood. I was at once alive to the position of things, which was fully verified by the application of my ear to the swelling; the *bruit de soufflet* was distinct without the stethescope—in fact, the lancet had entered into an aneurismal sac, which I only had the good fortune to avoid, by sticking it into the condensed fibrine, the walls of the sac. Having placed the finger upon the opening to restrain the hæmorrhage, I considered if the femoral artery could have been wounded by the lancet, but found that such could not possibly have been the case, for the opening was completely out of the line of that vessel; it was below it, and over the *adductor magnus*, while the blood was not propelled per saltum from the wound. I was determined to see the extent of the disease, consequently I introduced the same probe into the wound, and found that the aneurism extended to very nearly the same dimensions as the former abscess, from the groin to the ham. Having applied a compress and bandage, I at once pointed out to the friends of the patient the nature of the disease, and assured them that the only chance of saving the life of the boy was to tie the external iliac artery. The friends were at first annoyed at our mistake, and refused their consent; but, as I assured them the boy would bleed to death in a few days unless the operation was done, and that quickly, upon a slight hæmorrhage occurring, they changed their mind, and I was sent for to do the operation. Accordingly, on the fifth day after the opening, assisted by Dr. Corbin, I commenced the operation in the following way:—

Having placed the patient upon a table with his buttocks somewhat raised, I cut through the skin and areola tissue, commencing at about the outer pillar of the abdominal ring, going upwards and outwards, terminating about half-an-inch above the superior spinous process of the ilium, and an inch and a half inwards towards the median line. The aponeurisis of the external oblique was then divided upwards upon a director, the lower end of the oblique was then raised, the spermatic cord was drawn upwards and inwards with a blunt hook, the areola tissue covering the sheath of the vessels, which were both seen and felt pulsating, was slit up on a directory, the sheath itself was next opened, and an aneurismal needle armed with a ligature carefully placed under the artery. Having assured myself that the ligature was duly placed around the vessel, I tied it with a firm knot, when pulsation below the ligature immediately stopped. A suture was put into the divided edges of the wound, and this was duly supported with straps of sticking-plaster. The patient was put to bed, having borne the operation without an exclamation. After the operation, the limb became blue and cold; but by the application of temperate heat for two or three days, the limb completely regained its normal temperature, and the circulation was fully established by the anastomosing branches—no further bleeding occurred—the *bruit de soufflet* had subsided after the operation—and the wound granulated and healed, the ligature coming away about the fifteenth day. By degrees the swelling of the thigh subsided, the fluctuation gradually became more obscure, the skin returned to its natural color, and in the course of five or six months almost all the thickening and hardness had been dissipated, while the boy used the limb but with little less facility than the left; and, at the present time, even the slight halt in his gait has entirely subsided. Upon pressing in the groin, not the least pulsation was discoverable in the course of the femoral artery, while a cord-like feeling indicated that it was completely obliterated; and, save this symptom, and the scar made by the operation, not the slightest appearance of the disease is present in the limb.

The reflections which present themselves upon consideration of the case, are, first, as to the cause and nature of the aneurism; and, secondly, with regard to the operation which it was necessary to perform.

As a sequence of abscess, the aneurism was unique as to its character, and clearly occupied the whole extent of the seat of that disease; the femoral artery must, from the location of the abscess, have in all probability passed completely through its sac, and it must have influenced both that vessel and the profunda femoris in their course. I presume that the artery, in this instance, failed to be closed by the effusion of coagulable lymph along their course, as is usually the case under such circumstances; but being completely isolated, it died, the dead separated from the living parts, so that the patulous mouth of the vessel drove the blood into the old sac of the abscess, which became gradually distended with it, and became an extensive false aneurism; the previous thickening and consolidation of the areola tissue which formed the walls of the abscess, now became the sac of the aneurism, and prevented the blood being diffused through the limb, as in all probability would have been the case at the separation of the slough in the artery, had not the abscess preceded the eneurism, and under such circumstances would have had all the effect and character of traumatic diffused aneurism.

Again: As to the propriety of the operation performed, I think there could be no exception. Certainly, the rule in surgery, to tie both the extremities of the injured artery, so plainly and strenuously laid down by my venerated preceptor, G. J. Guthrie, Esq., did not hold good in this instance; although the facts in this case might have constituted a variety of traumatic aneurism, yet in all its conditions it bore a most natural resemblance to false aneurism. It was maintained by one of the first surgeons in Canada, that the seat of the aneurism should have been laid open, and the two extremeties of the artery tied, to ensure against all the chances of secondary hæmorrhage; such might possibly have been the rule, but it was certainly not admissible in this case. How could we be certain where we should find the bleeding vessels, and how many might have presented themselves? Picture to yourself the aneurismal sac laid open nearly from the groin to the ham, and after you had rolled out the vast clots of black blood, and come upon the thick fibrinous wall of the sac, many bleeding mouths might have presented themselves to your notice; perhaps the extremity of the femoral artery throwing out a powerful volume of blood—next, the patulous mouths of the profunda femoris, and perhaps many other branches of the main artery, sending a reflex current, all greatly enlarged, and now preparing to take on their anastomotic duty, as the main carriers of the blood and nourishers of the limb; and even did you find the bleeding mouths of the vessels, could you possibly have tied them? You would have been obliged to follow their course to where they were truly sound, before you could have put a ligature upon them. No, the idea was fearfully preposterous, and I am convinced, that the only resource was a ligature upon the external iliac; and even was this act contrary to a rule in surgery, but the end has sanctified the means, and points to a legitimate exception to that rule.

